# Best MRI sequences for identifying axillary lymph node markers in patients with metastatic breast cancer: an inter-reader observational study

**DOI:** 10.1186/s41747-020-00161-6

**Published:** 2020-06-12

**Authors:** Naziya Samreen, Asha A. Bhatt, Kalie Adler, Shannon Zingula, Katrina N. Glazebrook

**Affiliations:** 1 NYU Langone, 765 Stewart Ave, Garden City, NY 11530 USA; 2 Department of Radiology, Mayo Clinic, 200 1st street SW, Rochester, MN 55905 USA; 3 St. Vincent Healthcare, 2900 12th Ave N, Billings, MT 59101 USA

**Keywords:** Axilla, Breast neoplasms, Lymph nodes, Magnetic resonance imaging, Surgical instruments

## Abstract

**Background:**

We assessed confidence in visualization of markers within metastatic axillary lymph nodes (LNs) on magnetic resonance imaging (MRI), which were placed post-ultrasound (US)-guided biopsy.

**Methods:**

A retrospective review was performed on 55 MRI cases between May 2015 and October 2017. Twenty-two MRIs were performed before neoadjuvant therapy, and 33 MRIs were after its initiation. There were 34/55 HydroMARK®, 10/55 Tumark®, and 11/55 other marker types. Time interval between marker placement and MRI examination was 103 ± 81 days (mean ± standard deviation). Three readers with 1–30 years of experience independently assessed four axial sequences: unenhanced fat-suppressed three-dimensional T1-weighted spoiled gradient-recalled (SPGR), first contrast-enhanced fat-suppressed SPGR, T2-weighted water-only fast spin-echo (T2-WO), and T2-weighted fat-only fast-spin-echo (T2-FO).

**Results:**

Markers were 5.2× more likely to be visualized on T2-WO than on unenhanced images (*p* = < 0.001), and 3.3× more likely to be visualized on contrast-enhanced than on unenhanced sequences (*p* = 0.009). HydroMARK markers demonstrated a 3× more likelihood of being visualized than Tumark (*p* = 0.003). Markers were 8.4× more likely to be visualized within morphologically abnormal LNs (*p* < 0.001). After 250 days post-placement, confidence in marker brightness of HydroMARK markers on T2-WO images was less than 50% (*p* < 0.001). Inter-rater agreement was excellent for T2-WO and contrast-enhanced SPGR, good for unenhanced SPGR, and poor for T2-FO images.

**Conclusion:**

T2-WO and contrast-enhanced images should be used for marker identification. HydroMARK was the best visualized marker. Markers were easier to identify when placed in abnormal LNs. The visibility of HydroMARK markers was reduced with time.

## Key points

- T2-weighted water-only images have a higher probability of allowing marker identification compared to other imaging sequences.
- HydroMARK markers are more easily visualized on MRI compared to Tumark markers.
- Markers are easier to identify when the lymph node demonstrates abnormal morphology.

## Background

According to the National Comprehensive Cancer Network guidelines, systemic imaging should be considered in patients who are clinically stage IIB with advanced axillary disease, stage III, locally advanced, and in those with inflammatory breast cancer [1]. Magnetic resonance imaging (MRI) can help in the assessment of disease extent, including axillary lymphadenopathy, and change in tumor burden post-neoadjuvant therapy (NAT) [1, 2]. Assessment of breast cancer axillary lymph node (LN) metastases is critical for staging and surgical planning [3–7].

In patients with breast cancer who have suspicious axillary LNs, a marker is often placed within the LN at time of biopsy. Prior studies have established the importance of marker placement in the biopsy proven LN at diagnosis [8–12]. A marker is placed within suspicious or cytology/pathology proven metastatic LNs to facilitate localization and subsequent surgical removal. Hence, when these patients undergo MRI for staging or for assessment of response to NAT, the marker can be visualized on MRI. Thus, it is important to know which MRI sequences best demonstrate the marker because this helps identify the biopsy proven metastatic LN. Additionally, if the metastatic LN decreases in size on the post-NAT MRI, similar landmarks can be used to identify the marked metastatic LN to evaluate response to treatment.

The purpose of this study is to assess confidence in axillary LN marker visualization on four different MRI sequences to determine which sequence is the most useful in identification of the marker to help monitor response to NAT.

## Methods

After Institutional Review Board approval (Mayo Clinic Rochester, MN, October 17, 2016, ID 16-008036), a retrospective review was performed of all patients who underwent axillary LN biopsy with marker placement and subsequent breast MRI between May 2015 and October 2017 (29 months).

The two main markers used at our institution for axillary LN biopsies include the 18 gauge HydroMARK® markers (Mammotome, Cincinnati, OH) and Tumark® markers (Hologic, Bedford, MA). A HydroMARK marker has a biodegradable hydrogel polymer containing a central permanent metal marker. The hydrogel polymer immediately expands with deployment, containing > 90% water as hydration, which can last up to 12 months [13]. A Tumark marker consists of two parts: a metallic core made of titanium or stainless steel and a bioabsorbable suture-like netting that surrounds the metal [14].

All MRIs were performed at 1.5 Tesla (NY scanner, General Electric, Boston, MA) using a 7-channel dedicated bilateral breast coil. Axial, T1-weighted fat-suppressed three-dimensional spoiled gradient-recalled (SPGR) images were acquired before intravenous injection of gadolinium-based contrast agent and after intravenous injection of gadolinium-based contrast agent (Gadavist, 0.1 mmol/kg) at 1–1.5 min, 2–3 min, and about 7–8 min using a field of view of 28–40 cm, slice thickness 1.8–2.0 mm, in-plane resolution ≤ 1.0 mm^2^, repetition time 5.2 ms, echo time 2.5 ms, flip angle 10°, bandwidth 62.50 kHz, and phase direction left-to-right. Axial T2-weighted water-only (T2-WO) and fat-only (T2-FO) images were acquired using a fast spin-echo acquisition using a modified 3-point Dixon method for fat suppression (iterative decomposition of water and fat with echo asymmetry and least-squares estimation, IDEAL), field of view 28–40 cm, slice thickness 4.0 mm with 1.0 mm spacing, repetition time 4,505 ms, echo time 102 ms, echo-train length 14, matrix 320 × 224, number of excitations 2.0, and bandwidth 50.0 kHz.

Three readers with 1–30 year experience in breast MRI interpretation independently assessed for visualization of markers on the four sequences in the following order: (1) T2-WO, (2) T2-FO, (3) unenhanced SPGR, and (4) first contrast-enhanced SPGR. Readers were blinded to results from other readers and to the type of marker placed. An assessment was made by each reader whether the marker was visualized or not. Visualization of marker meant that the reader had > 50% confidence in marker visualization (noted as “high confidence”). Non-visualization of marker meant that the reader had < 50% confidence in marker visualization (noted as “low confidence”). Location of the marker (cortex or outside of the cortex) and LN morphology (normal *versus* abnormal) was also recorded. Criteria for abnormal lymph node morphology included focal or diffuse cortical thickness of greater than 3 mm or replacement of the normal fatty hilum. In addition, subset analysis for HydroMARK marker appearance on T2-WO sequence was recorded as dark or bright.

Date of marker placement, date of MRI, type of marker, and date of surgery were obtained from electronic medical records. It was also noted whether the MRI was before or after NAT. Results were correlated with both clinical history of marker placement and marker retrieval at surgical pathology.

Inter-rater agreement among the readers was calculated using the intraclass correlation co-efficient (ICC). Based on Koo and Li [15], the following scale was used to interpret the intraclass coefficient: < 0.40 = poor; 0.40–0.59 = fair; 0.60–0.74 = good; 0.75–1.00 = excellent [15]. Logistic regression was performed for predicting marker visualization according to marker type, LN morphology, location of marker, and change in HydroMARK marker brightness on T2-WO images with time. Odds ratio and confidence intervals were evaluated for each subset analysis. All statistical analyses were performed using the statistical toolbox for JMP 14 (SAS Institute Inc., Cary, North Carolina 27513, USA), and *p* values lower than 0.05 were considered statistically significant.

## Results

A total of 80 patients had a unilateral axillary LN marker placement during the 29-month period. Only 54 patients had an MRI after marker placement and were used in the analysis. The time interval between marker placement and MRI examination ranged from 0 to 366 days (mean 103 days; standard deviation 81 days). All the 54 patients had either pre-NAT or post-NAT MRI available for evaluation, except for one patient for whom both the pre-NAT and post-NAT MRI was available. Hence, a total of 55 MRIs were evaluated, 22 pre-NAT and 33 post-NAT. In the patient who had both pre and post-NAT MRI available, these were analyzed as separate studies with the readers blinded to the information that the study was from the same patient. In the post-NAT patients, the first MRI post-initiation of NAT was evaluated.

Of 55 patients, 27 went on to axillary LN dissection. Thirty-one patients underwent sentinel LN biopsy. Of those, 25 had no positive LNs, 4 had one positive LN, and 2 had two positive LNs. Three patients with positive sentinel LN biopsy went on to complete axillary LN dissection. A total of 27 patients underwent axillary LN dissection. A range of 0–7 positive LNs were identified at final surgical pathology in patients who underwent axillary LN dissection.

### Marker visualization by marker type

**Fig. 1:**
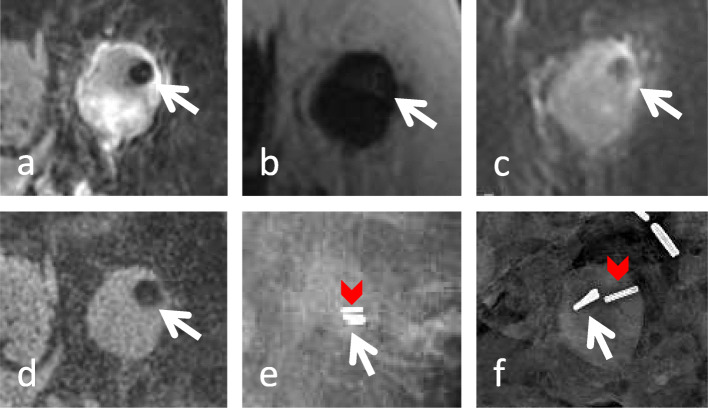
A 55-year-old female with left breast multifocal invasive ductal carcinoma and biopsy proven metastatic axillary lymphadenopathy. A MammoMark® (Mammotome, Cincinnati, OH) U-shaped marker was placed after biopsy of an abnormally enlarged axillary lymph node. Initial staging magnetic resonance imaging performed 1 week after marker placement demonstrates susceptibility artifact from the marker within an abnormally enlarged lymph node on various sequences. **a** On the T2-weighted water-only image, the marker is dark and seen with high confidence. **b** On the T2-weighted fat-only image, the marker is seen with low confidence (arrow). **c** On the first contrast-enhanced fat-suppressed three-dimensional T1-weighted spoiled gradient-recalled image, the marker is dark (arrow) and seen with high confidence. **d** On the unenhanced fat-suppressed three-dimensional T1-weighted spoiled gradient-recalled image, the marker is dark (arrow) and seen with high confidence. **e** Post-seed localization mammogram demonstrates the marker (arrow) adjacent to a radioactive seed (arrowhead/chevron). **f** Surgical specimen shows marker (arrow) and radioactive seed (arrowhead/chevron) within the metastatic lymph node

**Fig. 2:**
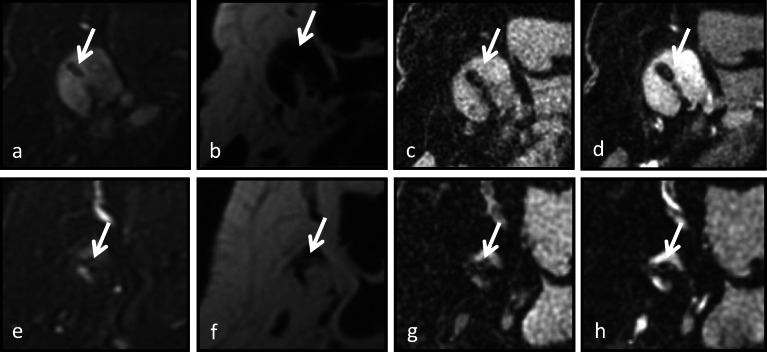
A 49-year-old female with invasive lobular carcinoma of the right breast with biopsy proven right axillary metastatic lymphadenopathy. A Tumark x-shaped marker was placed at site of axillary lymph node biopsy. **a**–**d** Initial staging magnetic resonance imaging (MRI) 2 weeks after lymph node marker placement. The marker is seen with high confidence on the T2-weighted water-only fast spin-echo image (**a**), the unenhanced fat-suppressed three-dimensional T1-weighted spoiled gradient-recalled image (**c**), and the first contrast-enhanced fat-suppressed three-dimensional T1-weighted spoiled gradient-recalled image (**d**), while it is seen with low confidence on the T2-weigheted fat-only fast spin-echo image (**b**). In the follow-up of neoadjuvant therapy, MRI was performed 6 months after biopsy marker placement. The marker is seen with low confidence on all images: T2-weighted water-only fast spin-echo image (**e**), T2-weighted fat-only fast spin-echo image (**f**), unenhanced fat-suppressed three-dimensional T1-weighted spoiled gradient-recalled image (**g**), and first contrast-enhanced fat-suppressed three-dimensional T1-weighted spoiled gradient-recalled image (**h**), with poor visualization attributed to the decreased size of lymph node cortical thickness

Of 55 markers, 34 were 18 gauge HydroMARK (Mammotome, Cincinnati, OH, USA), 10 were Tumark Professional (Hologic, Bedford, MA, USA), and 11 were “other” markers including Bard® wing-shaped marker (*n* = 2), Bard® ring-shaped marker (*n* = 2), Bard® ribbon-shaped marker (*n* = 2), Bard® M-shaped marker (*n* = 1), Senomark® coil-shaped marker (*n* = 1), Mammotome® barrel-shaped marker (*n* = 1), Hologic® stoplight-shaped marker (*n* = 1), and MammoMark® U-shaped marker (*n* = 1). An example of MRI imaging of U-shaped marker is provided in Fig. 1. Calculation of odds ratios was performed on all sequences/images according to marker type. The HydroMARK marker demonstrated a 3× higher likelihood of being visualized compared to the Tumark (*p* = 0.003). “Other” markers had a 3.3× more likelihood of being visualized than Tumark (*p* = 0.040). The odds ratios for the visualization of various markers are presented in Table 1. A representative case with a Tumark marker is presented in Fig. 2.

**Table 1:** Odds ratios for the visualization of various marker types with high confidence

Marker type 1/marker type 2	Odds ratio	*p* value	Lower limit of confidence interval	Upper limit of confidence interval
Tumark/HydroMARK	0.3	0.030*	0.120	0.896
Other markers/HydroMARK	1.1	0.857	0.514	2.226
Other markers/Tumark	3.3	0.040*	1.054	10.129
HydroMARK/Tumark	3.1	0.030*	1.116	8.352
HydroMARK/Other markers	0.9	0.857	0.449	1.945
Tumark/Other markers	0.3	0.040*	0.099	0.949

*Statistically significant

### Marker visualization by type of sequence/image

Of the 55 cases, marker visualization with high confidence among all readers was noted in 28 cases on T2-WO images, in 22 cases on the first contrast-enhanced SPGR images, in 9 cases on unenhanced SPGR images, and in only 1 case on T2-FO images. Evaluation of odds ratios performed for all the markers demonstrated the following: a marker was 3.3× more likely to be seen on the first contrast-enhanced SPGR sequence compared to the unenhanced SPGR sequence (*p* = 0.009). A marker was 5.2× more likely to be seen on the T2-WO sequence compared to the unenhanced SPGR sequence (*p* < 0.001) and 1.6× more likely to be seen on T2-WO sequence compared to the first contrast-enhanced SPGR sequence (*p* = 0.252). The odds ratios of the various sequences are summarized in Table 2.

**Table 2:** Odds ratios for the visualization of markers on the different images evaluated

Sequence 1/sequence 2	Odds ratio	*p* value	Lower limit of confidence interval	Lower limit of confidence interval
T2-FO/CE-SPGR	0.0	0.001*	0.004	0.216
T2-FO/SPGR	0.1	0.027*	0.011	0.759
CE-SPGR/SPGR	3.3	0.009*	1.360	8.168
T2-FO/T2-WO	0.0	< 0.001*	0.002	0.138
CE-SPGR/T2-WO	0.6	0.252	0.302	1.368
SPGR/T2-WO	0.2	< 0.001*	0.079	0.470
CE-SPGR/T2-FO	36	0.001*	4.633	279.721
SPGR/T2-FO	10.8	0.027*	1.318	88.506
SPGR/CE-SPGR	0.3	0.009*	0.122	0.735
T2-WO/T2-FO	56	< 0.001*	7.228	433.896
T2-WO/CE-SPGR	1.6	0.252	0.731	3.3106
T2-WO/SPGR	5.2	< 0.001*	2.130	12.624

*CE-SPGR* First contrast-enhanced fat-suppressed three-dimensional T1-weighted spoiled gradient-recalled images, *SPGR* Unenhanced fat-suppressed three-dimensional T1-weighted spoiled gradient-recalled images, *T2-FO* T2-weighted fat-only fast-spin-echo images, *T2-WO* T2-weighted water-only fast spin-echo images. *Statistically significant

**Fig. 3:**
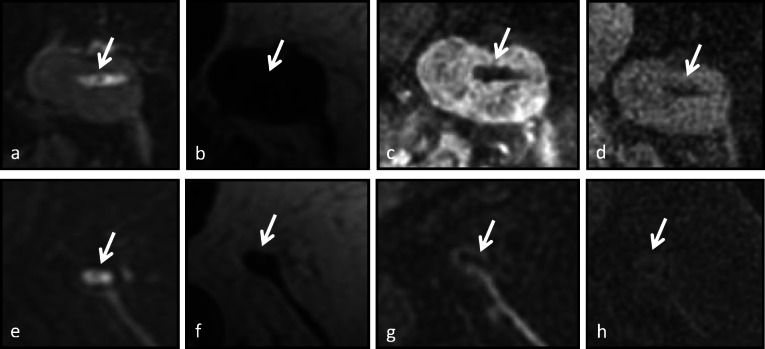
A 59-year-old female with newly diagnosed left breast invasive ductal carcinoma grade 3 with known metastatic left axillary lymphadenopathy. **a**–**d** Initial staging magnetic resonance imaging (MRI) performed 21 days after HydroMARK placement. The marker (arrow) is seen with high confidence on the T2-weigheted water-only fast spin-echo image (**a**), the unenhanced fat-suppressed three-dimensional T1-weighted spoiled gradient-recalled image (**c**), and the first contrast-enhanced fat-suppressed three-dimensional T1-weighted spoiled gradient-recalled image (**d**), while it is seen with low confidence on the T2-weigheted fat-only fast spin-echo image (**b**). **e**–**h** NAT follow-up MRI performed approximately 6 months after marker placement shows decreased size of lymph node. After NAT, the marker (arrow) is seen with high confidence on the T2-weigheted water-only fast spin-echo image (**e**) and the first contrast-enhanced fat-suppressed three-dimensional T1-weighted spoiled gradient-recalled image (**h**) sequences, while it is seen with low confidence visibility on the T2-weigheted fat-only fast spin-echo image (**f**) and the unenhanced fat-suppressed three-dimensional T1-weighted spoiled gradient-recalled image (**g**). Note, the decreased T2 brightness of the marker gel content 6 months after placement (**e**) compared to the initial MRI after placement (**a**)

A subset analysis was performed for visualization of the HydroMARK marker, since this was the majority of marker type (*n* = 34). Marker visualization with high confidence among all readers was noted in 17 cases on T2-WO images, in 10 cases on the first contrast-enhanced SPGR images, in 3 cases on unenhanced SPGR images, and zero cases on T2-FO images. Odds ratios for visualization of the HydroMARK marker were calculated. First contrast-enhanced SPGR sequence was 6.4× more likely to show the HydroMARK marker compared to pre unenhanced SPGR sequence (*p* = 0.003). The HydroMARK marker was 11.7× more likely to be seen on the T2-WO sequence compared to the unenhanced SPGR sequence (*p* < 0.001) and 1.8× more likely to be seen on the T2-WO sequence compared to the first contrast-enhanced SPGR sequence, but these results were not statistically significant (*p* = 0.225). The HydroMARK marker was not seen with high confidence on the T2-FO sequence. A representative case with a HydroMARK marker is shown in Fig. 3.

### Marker appearance on T2-WO images

**Fig. 4:**
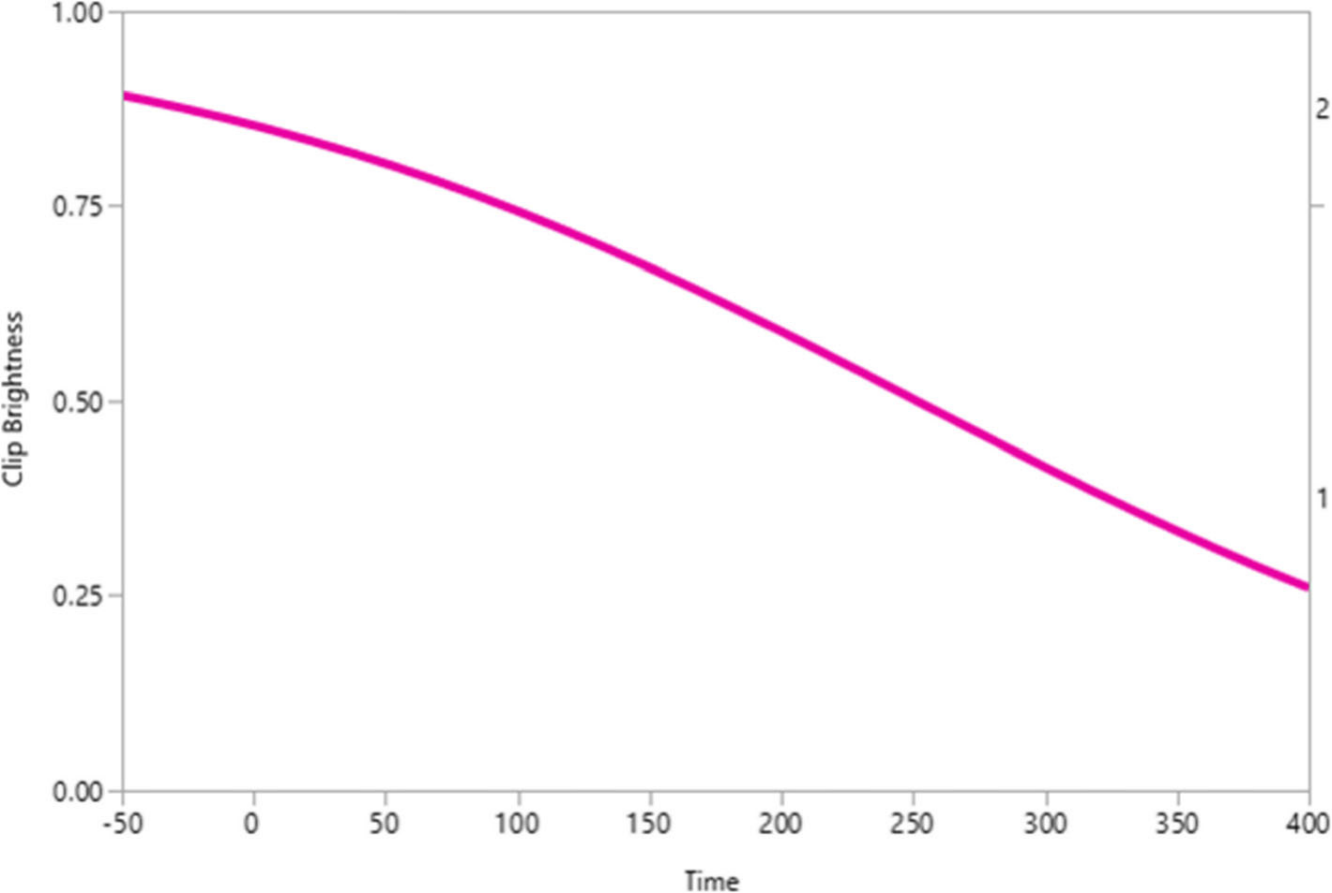
Plot for marker brightness *versus* time (days). Data are referred to HydroMARK marker on T2-weighted water-only fast spin-echo images

Overall, of the 43 markers seen with high confidence on the T2-WO images, 24 were hyperintense and 19 were hypointense. Of the 28 HydroMARK markers seen with high confidence on the T2-WO images, 21 were hyperintense and 7 were hypointense. Of the 6 Tumark markers seen with high confidence on the T2-WO images, 2 were hyperintense and 4 were hypointense. Of the 9 “other” markers seen with high confidence on the T2-WO images, 1 was hyperintense and 8 were hypointense. HydroMARK marker brightness on the T2-WO images specifically decreased with time. After 250 days, confidence in HydroMARK marker brightness was less than 50% (*p* < 0.001). These findings are presented in Fig. 4.

### Marker location

Of the total 55 markers, 40 were noted to be within the cortex, while the remaining 15 were noted to be outside the cortex (within hilum or surrounding tissues). A marker was more 1.2× more likely to be visualized if placed in the cortex *versus* out of the cortex, but the results were not statistically significant (*p* = 0.625).

### LN morphology

Of the 55 lymph nodes with marker placement, 26 were categorized as normal morphology, and 29 lymph nodes were categorized as abnormal in our study. A marker was 8.4× more likely to be visualized if the node was abnormal in size/morphology (*p* < 0.001). A representative case from a marker belonging to the “other” category placed within an abnormal LN is shown in Fig. 1.

### Inter-reader agreement

Inter-rater agreement was excellent for T2-WO images (single measure ICC 0.76), excellent for first contrast-enhanced SPGR images (single measure ICC 0.76), good for unenhanced SPGR images (single measure ICC 0.60), and poor for the T2-FO images (single measure ICC 0.24).

## Discussion

Although localization of axillary LN markers is typically not performed with MRI, identification of pathologic axillary LNs on MRI is very important in the staging and treatment of breast cancer. Many LNs decrease in size after NAT making it harder to identify which LN was metastatic, and in such cases, the marker can be used as a point of reference to monitor response to NAT. There is not much data on MRI sequences that are best to identify biopsy markers within axillary LNs. Multiple studies, however, have evaluated MRI characteristics that help identify pathologic LNs [16–22]. A systematic review demonstrated that a protocol with unenhanced T1- and T2-weighted with ultra-small iron particles-enhanced T2*-weighted sequences and a dedicated axillary protocol was most promising in identifying metastatic axillary LNs [23]. The negative predictive value of such a protocol was found to be similar to that of sentinel LN biopsy. It was also suggested that a dedicated axillary protocol, for example, one that uses a radiofrequency coil placed on the axilla, was better than a more standard protocol covering the breast and axilla in the same field of view [23].

In our study, we looked at various factors affecting marker visibility in patients receiving or not receiving NAT. The T2-WO and contrast-enhanced SPGR images overall demonstrated the best likelihood of marker visualization. It was also noted that contrast-enhanced images were better to identify markers compared to unenhanced images, and that markers placed within morphologically abnormal LNs were better visualized. These findings are explained by the dark signal void (susceptibility artifact) caused by the biopsy marker. The dark susceptibility artifact makes the marker easy to identify within the hyperintense LN cortex on T2-WO, and within the homogenous enhancement of the LN cortex on post-contrast sequences when the marker was placed in the cortex, especially when the cortex is morphologically enlarged. When markers are placed in the hilum, the signal void from the marker can sometimes be difficult to separate from the surrounding suppressed fat which also appears hypointense on MRI.

In our study, HydroMARK markers were significantly better visualized on MRI compared to Tumark markers, which may be a consideration during marker placement within metastatic axillary LNs. In axillary LNs containing the HydroMARK marker, the gel within the marker was easily identifiable on T2-WO images due to its hyperintense signal from the water content. Our study also demonstrated that the brightness within this marker decreased with time as expected, and after 250 days, confidence in identifying marker brightness was less than 50% for the HydroMARK marker. This is secondary to the gel content within the HydroMARK marker gradually being resorbed with time [13].

Once the marker is identified within the metastatic LN, even if the LN decreases in size after NAT, the anatomical landmarks surrounding the metastatic node may be used to assess response to NAT on MRI. For example, it can be noted if the marker is within the high axilla or low axilla, and other adjacent LNs and surrounding vessels can be used as landmarks to help determine the marked node on subsequent MRIs after NAT, even if the marker is less conspicuous with time. We found the T2-FO images to be very poor in identification of the biopsy marker for all types, as a lot of artifact was noted at the fat/water interfaces, making it difficult to separate from the biopsy marker. There was no statistically significant difference between markers placed in the cortex and outside of the cortex in our study but abnormal LN morphology did make it easier to identify the marker.

The inter-reader agreement between the three readers was overall good or excellent in our study (with the only exception of T2-FO images), adding more confidence into our results.

Limitations of this study include that this is a retrospective review performed at a single institution with a relatively small sample size. Larger studies would help increase the confidence in our results, although the number of our patients was enough to reach statistical significance. Additionally, it is possible that artifact could have been mistaken for a marker within the axillary nodes. However, this is why the confidence scale was set to less than 50% for non-visualization of marker; therefore, the authors had to be > 50% confident that this was indeed a marker and not an artifact. Our study is also limited by the number of various markers types that were included, as we had more HydroMARK markers compared to Tumark and markers in the “other” category. Moreover, images were reviewed in the order that they had been acquired (T2-WO, T2-FO, unenhanced SPGR, and contrast-enhanced SPGR). This may have introduced some bias, as knowledge from one sequence may have influenced marker evaluation on the next sequence from the same patient. However, once a sequence had been assessed, the readers were not allowed to change their results or go back to a previously evaluated sequence to compare it to the next. Another limitation is that only one patient within this study had both before- and after-NAT MRI, and both MRIs were used independently for analysis, with readers being blinded to the information that it was the same patient. It would be interesting to evaluate the appearance on pre- and post-NAT MRIs on subsequent studies. Finally, we only evaluated sequences that are specific to our General Electric MRI scanner and protocol. Other sequences that may be used at other institutions such as STIR, in phase non-fat saturated, and spectrally fat-suppressed T2-weighted sequences were not evaluated.

In conclusion, our study showed that axillary LN markers are best seen on T2-WO and contrast-enhanced SPGR images, and the HydroMARK was better visualized compared to Tumark. In addition, we found that markers were easier to identify when placed in abnormal LNs and that the visibility of HydroMARK marker was reduced with time, likely due to its gel content being resorbed.

## Data Availability

The datasets used and/or analyzed during the current study are available from the corresponding author on reasonable request.

## Abbreviations

ICC: Intraclass correlation coefficient
LN: Lymph node
MRI: Magnetic resonance imaging
NAT: Neoadjuvant therapy
SPGR: Spoiled gradient-recalled
T2-FO: T2-weighted fat-only fast-spin-echo
T2-WO: T2-weighted water-only fast spin-echo
