# Correction to: Best MRI sequences for identifying axillary lymph node markers in patients with metastatic breast cancer: an inter-reader observational study

**DOI:** 10.1186/s41747-020-00181-2

**Published:** 2020-08-18

**Authors:** Naziya Samreen, Asha A. Bhatt, Kalie Adler, Shannon Zingula, Katrina N. Glazebrook

**Affiliations:** 1 NYU Langone, 765 Stewart Ave, Garden City, NY 11530 USA; 2 Department of Radiology, Mayo Clinic, 200 1st street SW, Rochester, MN 55905 USA; 3 St. Vincent Healthcare, 2900 12th Ave N, Billings, MT 59101 USA

**Correction to: Eur Radiol Exp 4, 34 (2020)**

**https://doi.org/10.1186/s41747-020-00161-6**

The original article [1] contains an error in the second paragraph of the **Methods** section regarding the description of the composition of a Tumark marker.

The original article states:

*“A Tumark marker consists of two parts: a metallic core made of titanium or stainless steel and a bioabsorbable suture-like netting that surrounds the metal…”*

This statement should be disregarded in favour of the following statement:

*“A Tumark marker is made of non-resorbing nitinol that expands into a distinct shape upon deployment…”*
